# Activation of Autophagy Contributes to Sevoflurane-Induced Neurotoxicity in Fetal Rats

**DOI:** 10.3389/fnmol.2017.00432

**Published:** 2017-12-22

**Authors:** Xingyue Li, Ziyi Wu, Yi Zhang, Ying Xu, Guang Han, Ping Zhao

**Affiliations:** Department of Anesthesiology, Shengjing Hospital, China Medical University, Shenyang, China

**Keywords:** sevoflurane, midtrimester, cognitive impairment, neural stem cells, neurotoxicity, autophagy, apoptosis, neurogenesis

## Abstract

Numerous animal studies have demonstrated that commonly used general anesthetics may result in cognitive impairment in the immature brain. The prevailing theory is that general anesthetics could induce developmental neurotoxicity via enhanced apoptosis. In addition, inhibited proliferation induced by anesthetics has also been reported. So far, whether autophagy, a well-conserved cellular process that is critical for cell fate, also participates in anesthesia-induced neurotoxicity remains elusive. Here, we first examined autophagy-related changes after sevoflurane exposure and the effect of autophagy on apoptosis and proliferation, and we also explored the underlying mechanisms of autophagy activation. Pregnant rats were exposed to 2 or 3.5% sevoflurane for 2 h on gestational day 14 (G14); then, markers of autophagy and expression of autophagy pathway components were measured in fetal brains 2, 12, 24, and 48 h after anesthesia. Changes in neural stem cell (NSC) apoptosis, neurogenesis, neuron quantity and learning and memory function were examined after administration of an autophagy or PTEN inhibitor. The expression of microtubule-associated protein 1 light chain 3 (LC3)-II, Beclin-1 and phosphatase and tensin homolog on chromosome 10 (PTEN) were increased in the 3.5% sevoflurane group, while Sequestosome 1 (P62/SQSTM1), phospho-protein kinase B/protein kinase B (p-Akt/Akt) and mammalian target of rapamycin (mTOR) were decreased. 3-methyladenine (3-MA), an inhibitor of autophagy, or dipotassium bisperoxo-(5-hydroxypyridine-2-carboxyl)-oxovanadate (V) (bpV), a PTEN inhibitor, significantly attenuated the activation of autophagy, reversed the decreased expression of B-cell lymphoma-2 (Bcl-2) and reduced the number of terminal-deoxynucleoitidyl transferase mediated nick end labeling (TUNEL) positive cells, ameliorated the decline of Nestin expression, Ki67 positive cell rate, neuron quantity and cross platform times, and shortened the prolonged escape latency. Our results demonstrated that 2 h 3.5% sevoflurane exposure at G14 induced excessive autophagy in the fetal brain via the PTEN/Akt/mTOR pathway. Autophagy inhibition reversed anesthesia-induced NSC apoptosis, proliferation decline and memory deficits.

## Introduction

Advanced surgical technologies have made it possible to conduct more fetal intervention operations. And most of them are carried out in the second trimester, which is regarded as a relatively safe period compared with earlier or later gestation. However, the fetal brain is still sensitive to changes in the external environment during this period.

Mammalian neural stem cells (NSCs) have attracted much attention as they play a significant role in brain development (Chung and Yu, 2013). Owing to their ability to proliferate and to differentiate into different neural lineages, NSCs determine the formation and function of both embryonic and adult mammalian brains. The main content of the nervous system development in the second trimester is NSC proliferation (Palanisamy, 2012). However, our knowledge about the effect of anesthetics on the NSCs is very limited.

A large body of animal research has demonstrated that use of common general anesthetics may result in cognitive impairment in immature brains (Jevtovic-Todorovic et al., 2003; Stratmann et al., 2009; Palanisamy et al., 2011; Fang et al., 2012). Most studies were conducted during the critical phase of synapse formation, which occurs postnatally in rodents (Palanisamy, 2012). Drugs used for general anesthesia can induce developmental neurotoxicity via apoptosis, as demonstrated in diverse animal models (Kong et al., 2012a; Olutoye et al., 2016; Yu et al., 2017). Increasing evidence suggests that anesthesia exposure in the second trimester may also lead to adverse consequences for the offspring (Kong et al., 2011, 2012b; Zheng et al., 2013). According to our unpublished data, sevoflurane, the most commonly used inhalation anesthetics, can lead to increased NSC apoptosis, reduced proliferation and offspring’s learning impairment at high dose (3.5%) as compared to either lower concentration (2%) or control group (30% oxygen); no difference was observed in the low concentration and control group. The results of the study by Wang et al. also suggested that exposure of G14 rats to 3 or 4% sevoflurane anesthesia showed a negative effect on the learning and memory abilities of their offspring, and both inhibited proliferation and increased apoptosis of nerve cells were revealed (Wang et al., 2017).

Autophagy is a highly conservative, self-degradative process which is involved in both physiological and pathological situations, such as nutrient starvation, stress, aging, and intracellular infection (Yang and Klionsky, 2010). Basal levels of autophagy can degrade damaged organelles and long-live proteins; by contrast, overactive autophagy may interfere with physiological process in the cell, even lead to cell death (Klionsky and Emr, 2000; Komatsu et al., 2006; Mukhopadhyay et al., 2014). It has been reported that ethanol can trigger the activation of autophagy in the developing brain (Chen et al., 2012). Since ethanol and sevoflurane are both GABA_A_ agonists, we hypothesized that sevoflurane exposure may also increase autophagy level in fetal brain. In addition, emerging evidence indicates that, not only is autophagy related to cell proliferation, there are also complex interactions between autophagy and apoptosis (Wirawan et al., 2012). How autophagy activation affects these processes was investigated in the current study.

To gain insight into the role of autophagy in the anesthestic neurotoxicity during the early stage of brain development, we utilized the embryonic day 14 (E14) rat model since the mid-pregnancy is considered safe for fetal intervention (Palanisamy, 2012). We hypothesized that 3.5% sevoflurane could lead to overactive autophagy, which is responsible for the increased apoptosis, decreased proliferation and impaired neurocognitive function during this period of pregnancy.

## Materials and Methods

### Animals

All animal experiments were conducted following the National Institute of Health Guideline for the Care and Use of Laboratory Animals. This study was approved by the Institutional Animal Care and Use Committee of Shengjing Hospital, China Medical University (No. 2015PS211K).

Two-month-old female Sprague-Dawley rats (220–250 g) and male SD rats (250–280 g) used in the study were procured from Research and Development Center of Shengjing Hospital. The rats were housed at a constant temperature of 22 ± 1°C with free access to water and food. They were maintained under a 12 h light/12 h dark cycle (lights-on from 7 AM to 7 PM). Each male rat was put in the cage together with 3–4 female rats. The next morning, male rats were removed, and vaginal smear of female rats were conducted. If sperm was observed, the female rat was considered pregnant and the day was recorded as gastation day 0 (G0) with respect to the pregnant rats and embryonic day 0 (E0) with respect to the fetus.

### Sevoflurane Exposure

Rats were put into a acrylic chamber for anesthesia with two holes on the opposite sides of the chamber, each connected to a duct. One duct was connected to a sevoflurane vaporizer transporting mixed gas including oxygen, nitrogen and sevoflurane; the other duct was used for transporting the gas samples to the monitor. The temperature in the chamber was maintained via heating pool. In the control (CON) group, rats were placed in a chamber which was ventilated with 30% oxygen at a flow rate of 2 L/min for 2 h. 2% sevoflurane (2%SEV) group and 3.5% sevoflurane (3.5%SEV) group received 2 or 3.5% sevoflurane for 2 h with 30% oxygen. The rats breathed spontaneously during the gas exposure. When the anesthesia was terminated, the rats were transferred into the normal cages, and the fetuses in the uterus were obtained through cesarean section 2, 12, 24, and 48 h after gas exposure or natural birth, respectively.

### Drug Administration

Rats in 3.5% sevoflurane plus 3-Methyladenine group (3.5%SEV + 3-MA) were intraperitoneally injected with 3-MA (40 mg/kg), and rats in 3.5% sevoflurane plus bpV group (3.5%SEV+bpV) were intraperitoneally injected with bpV (0.2 mg/kg). Drug administration mentioned above were all initially conducted 1 h before anesthesia. Other groups that were compared with the two groups above were treated with the same volume of vehicle solution.

### Transmission Electron Microscopy

G14 rats anesthetized with pentobarbital 2 h after 3.5% sevoflurane exposure were transcardially given 200mL normal saline followed by 200 mL 4% paraformaldehyde. The immersion fixation of tissue samples (1 mm^3^) from the fetal brain was completed in the 2% glutaraldehyde and the samples were post-fixed in 1% osmium tetraoxide for 2 h. Then the tissues were infiltrated in a mixture of acetone and resin before undergoing dehydration with graded ethanol. The tissue samples were then embedded in resin. Sections were obtained and followed by staining of 2% uranyl acetate and lead citrate. Autophagic specific structures in the NSCs were observed.

### Western Blot

The whole brains of fetuses were removed immediately when the female rats were under cesarean section, and stored at -80°C before use. The samples were homogenzied and ultrasonicated in radio-immunoprecipitation assay buffer (P1103B; Beyotime, China) containing phenylmethylsulfonyl fluoride solution (ST506; Beyotime, China). The homogenate was centrifuged at 1200 g for 30 min, and the supernatant was collected then measured by BCA Protein Assay Kit (P0010; Beyotime, China) in order for equal concentration. Forty micrograms of protein per lane was separated by electrophoresis using 10 or 12.5% SDS-polyacrylamide gel and then electrotransferred to polyvinylidene fluoride members (IPVH0010; Millipore, Germany). The membranes were blocked in 5% non-fat milk or BSA and incubated with primary antibodies overnight at 4°C. The next day, the membranes were incubated with the second antigen-antibodies at room temperature (RT) for 2 h. Proteins were detected and photographed by GE Amersham Imager 600 using SuperSignal^®^ West Pico Chemiluminescent Substrate (34080; Thermo, United States). The primary antibodies were: LC3B Anitibody (1:1000, 2775; Cell Signaling Technology, United States), Beclin-1 Antibody (1:1000, 3738; Cell Signaling Technology, United States), P62/SQSTM1 Antibody (1:2000, P0067; Sigma–Aldrich, United States), PTEN Antibody (1:1000, 9188; Cell Signaling Technology, United States), Akt Antibody (1:1000, 9272; Cell Signaling Technology, United States), Phospho-Akt Antibody (1:1000, 4060; Cell Signaling Technology, United States), mTOR Antibody (1:1000, 2983; Cell Signaling Technology, United States), Nestin Antibody (1:1000, MAB353; Millipore, Germany), Bcl-2 Antibody (1:1000, 12789-1-AP; Proteintech, United States). Number of rats per group in Western blot experiment was 5 (*n* = 5).

### Immunohistochemistry

Whole brains were removed from fetuses. Fetal brains were first immersed in 4% PFA at 4°C for 24 h, then embedded in paraffin after dehydration in graded ethanol. Transverse plane sections (2.5 μm, the thickness of following brain sections is all the same) were deparaffinized and heated in citrate buffer at 121°C for 7 min. PBS containing 10% fetal bovine serum and 3% hydrogen peroxide was added to each section to reduce background staining. Next, sections were incubated with the Nestin Antibody (1:200, MAB353; Millipore, Germany) or NeuN Antibody (1:200, MAB377; Millipore, Germany) at 4°C for 12 h in a humidified chamber. After three 5 min rinses in PBS, the sections were incubated with peroxidase-conjugated secondary antibody and DAB was applied for chromogenic staining. The nuclei were stained with hematoxylin. Finally the sections were photographed with Nikon C1 microscope by an investigator who was blinded to the experimental intervention. Each brain section of Nestin staining was photographed at least 2 random fields of view and OD value was quantified by NIS-Elements AR Analysis 4.50.00 software. We sliced each brain tissue serially and selected 3 comparable brain slices per rat for NeuN staining. NeuN-positive cells were counted in a random reticle (approximately 0.01 mm^2^) at 400× magnification. Average values of three determinations were converted to the ratio of the CON group values and used to calculate the relative neuron quantities. Number of rats per group in immunohistochemistry experiment was 5 (*n* = 5).

### Immunofluorescence Staining

The brain sections were deparaffinized and heated in citrate buffer for antigen retrieval. PBS containing 10% fetal bovine serum was added to each section to reduce background staining for 30 min at room temperature. Sections were incubated with two kinds of primary antibodies simultaneously at 4°C overnight in a humidified chamber for immunofluorescence double staining, and then they were incubated with secondary antibodies for 2 h at RT. When performing the immunofluorescence staining for Ki67, PBS with 0.3% Triton X-100 was used to rinse the sections instead of PBS. The antibodies used were: LC3B Antibody (1:200, ab48394; Abcam, United Kingdom), Nestin Antibody (1:200, MAB353; Millipore, Germany) and Ki67 Antibody (1:200, YM3064; ImmunoWay, United States). The nuclei were stained with DAPI. Sections were photographed with Nikon C1 microscope by an investigator who was blinded to the experimental interventions. Each Ki67 staining section with 1–2 random fields of view was photographed, and the numbers of Ki67 positive cells and total cells were counted at 400× magnification by NIS-Elements AR Analysis 4.50.00 software, then we calculated the ratio of the number of Ki67 positive cells to the total number of cells. Number of rats per group in immunofluorescence staining experiment was 5 (*n* = 5).

### TUNEL and Immunofluorescence Double Staining

The brain sections were deparaffinized and heated in citrate buffer for antigen retrieval. PBS containing 10% fetal bovine serum was added to each section at room temperature for 30 min to reduce background staining. The sections were incubated with the Nestin Antibody (1:200, MAB353; Millipore, United States) at 4°C overnight in a humidified chamber and then incubated with secondary antibody and terminal deoxynucleotidyl transferase (TdT) and dUTP (11684817910; Roche, Switzerland) simultaneously at 37°C for 1 h. Nuclei were stained with DAPI. Each brain section with 1 random field of view was photographed with Nikon C1 microscope by an investigator who was blinded to the experimental interventions. The number of TUNEL/Nestin-positive cells was counted at 400× magnification by NIS-Elements AR Analysis 4.50.00 software. Number of rats per group in TUNEL experiment was 5 (*n* = 5).

### Morris Water Maze

To test the spatial learning and memory ability of the rat offspring, Morris water maze (MWM) experiments were conducted 28–33 days after birth. The MWM was performed in a circular pool with black walls (diameter: 160 cm, depth: 60 cm). The pool was filled with 30-cm-deep water at 20°C. An escape platform (diameter: 12 cm) was located 1.5 cm below the surface of water and at the center of the target quadrant. Probe trial sessions began at 8: 00 am for 5 days and were conducted four times (once per quadrant) daily with 30 min rest time. Rats (*n* = 10, per group) were put into the water to search for the hidden platform from four quadrants facing the pool wall. The duration spent on finding the platform (escape latency) was within 90 s. When the rats did not find the platform within 90 s, they were guided toward the platform and the escape latency was recorded as 90 s. After each trail, rats were made to stand on the platform for 20 s. During spatial probe test, rats were permitted to swim for 90 s freely after the platform was taken away from the maze. The entire process of MWM experiment was recorded by a camera located above the pool and analyzed using image analysis software (Shanghai Mobiledatum Ltd., China).

### Statistical Analysis

The data were checked by Bartlett’s test for equal variances and Shapiro–Wilk test for normality. Parametric data of three groups were compared by one-way analysis of variance (ANOVA) followed by the Student–Newman–Keuls *post hoc* test, and non-parametric data of three groups were compared by Kruskal–Wallis with Dunn’s Multiple comparison test. The data of escape latency in the Morris water maze test were analyzed using two-way ANOVA (sevoflurane treatment as between-groups and days as repeated measures factors) followed by Bonferroni post test for the four groups’ comparisons. The spatial probe test data were analyzed using Kruskal–Wallis with Dunn’s Multiple comparison test. All the data were analyzed with GraphPad Prism 5.0 software or SPSS 20.0 software. Differences were considered to be statistically significant if *P* < 0.05. Data were presented as mean ± SD.

## Results

### 3.5% Sevoflurane Exposure Upregualtes Autophagy in NSCs

**FIGURE 1:**
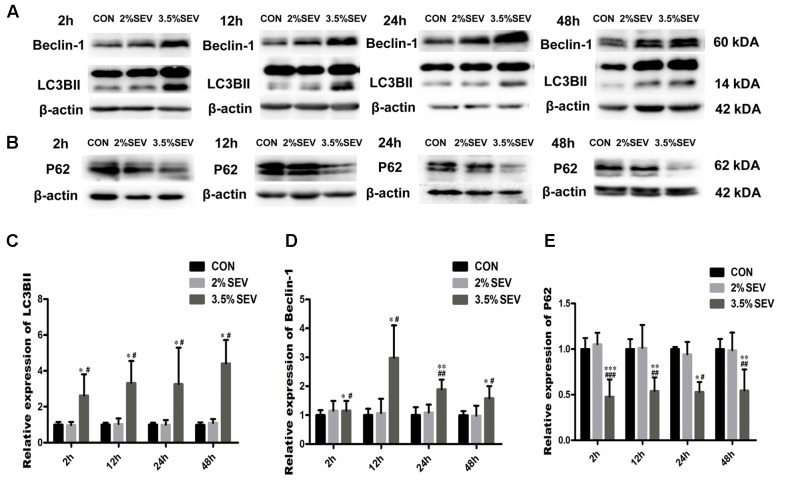
3.5% sevoflurane exposure upregualted autophagy in the NSCs. 2 h 3.5% sevoflurane exposure increased expressions of LC3BII and Beclin-1 **(A)**, and reduced the expression of P62 2, 12, 24, 48 h later **(B)**. Quantification of LC3BII **(C)**. Quantification of Beclin-1 **(D)**. Quantification of P62 **(E)**. Values are presented as mean ± SD, *n* = 5; *^∗^P* < 0.05, *^∗∗^P* < 0.01, compared with the CON group, ^#^*P* < 0.05, ^##^*P* < 0.01, compared with the 2%SEV group. One-way ANOVA with Newman–Keuls *post hoc* test or Kruskal–Wallis with Dunn’s Multiple comparison test was used for data analysis.

**FIGURE 2:**
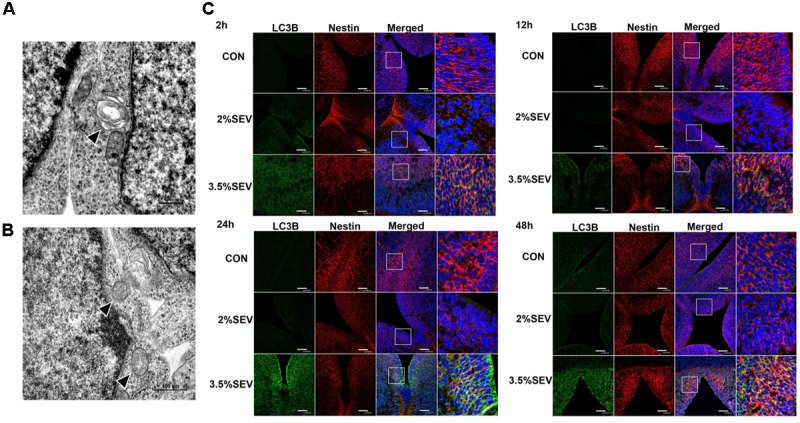
3.5% sevoflurane exposure upregualted autophagy in the NSCs. Effects of 3.5% sevoflurane on NSC autophagy in TEM scan. Autophasosome (arrow head in **A**) and autolysosome (arrow head in **B**) was observed after 2 h 3.5% sevoflurane exposure. Scale bar = 500 nm. Sevoflurane increased LC3B expression in NSCs 2, 12, 24, and 48 h after anesthesia **(C)**. LC3B (green) co-localized with Nestin (red) showed punctate pattern in the 3.5%SEV group, whereas both the CON group and 2%SEV group presented diffused green light labeling. Scale bar = 50 μm.

In our previous study, we found that 3.5% sevoflurane anesthesia resulted in acute neurotoxicity with learning and memory impairment in rat offsprings; whereas no differences were observed between rats in the 2% sevoflurane group and the control group. To further study the autophagic activity in fetal brains, we examined the expression of the key autophagy-related proteins including LC3BII, Beclin-1 and P62/SQSTM1 2, 12, 24, and 48 h after the exposure (**Figure 1**). LC3 has two forms: when autophagy is initiated, LC3II is formed by conjugating LC3I to phosphatidylethanolamine. Since the expression of LC3I is quite abundant in brain tissue (Yang et al., 2011), we examined the expression of LC3 with LC3BII compared to β-actin instead of the ratio of LC3II/LC3I. Beclin-1 is another well-studied autophagy-related protein that participates in the formation of autophagosomes. Yet measuring LC3BII and Beclin-1 can not provide a full picture of the extent of autophagy; thus we also examined the expression of P62/SQSTM1, which is specially degraded in autolysosomes, to detect whether the autophagic flux was intact. Our results revealed increased expression of LC3BII and Beclin-1, as well as decreased expression of P62 in the 3.5%SEV group as compared to the CON and 2%SEV group; no difference was observed between the 2%SEV and CON group (**Figure 1**, 3.5%SEV *vs.* CON: LC3BII: *P* < 0.05, *P* < 0.05, *P* < 0.05, *P* < 0.05; Beclin-1: *P* < 0.05, *P* < 0.05, *P* < 0.01, *P* < 0.05; P62: *P* < 0.001, *P* < 0.01, *P* < 0.05, *P* < 0.01; 3.5% SEV *vs.* 2%SEV: LC3BII: *P* < 0.05, *P* < 0.05, *P* < 0.05, *P* < 0.05; Beclin-1: *P* < 0.05, *P* < 0.05, *P* < 0.01, *P* < 0.05; P62: *P* < 0.001, *P* < 0.01, *P* < 0.05, *P* < 0.01; 2, 12, 24, and 48 h after anesthesia, respectively). To further verify these results, fetal brains were harvested 2 h after the 3.5% sevoflurane exposure and examined under the transmission electron microscope (TEM), which is considered as the gold standard for detecting the autophagic process. TEM revealed the presence of double-membrane vacuolar structures containing cytoplasmic content in the cytoplasm of NSCs, representing the formation of autophagosomes and autolysosomes (**Figures 2A,B**). To further verify autophagy induction in NSCs, we performed double immunostaining of LC3B and Nestin, which is a maker of NSCs. In the brain sections from the 3.5%SEV group, LC3B co-localized with Nestin presenting punctate pattern, whereas brain sections from both the CON group and 2%SEV group showed diffused green light labeling. Taken together, these results indicated that sevoflurane activated autophagy in NSCs after exposure to 3.5% sevoflurane; and there were no differences observed between the 2%SEV group and CON group (**Figure 2C**).

### Involvement of the PTEN/Akt/mTOR Signaling Pathway in the Autophagy Activation Induced by 3.5% Sevoflurane

**FIGURE 3:**
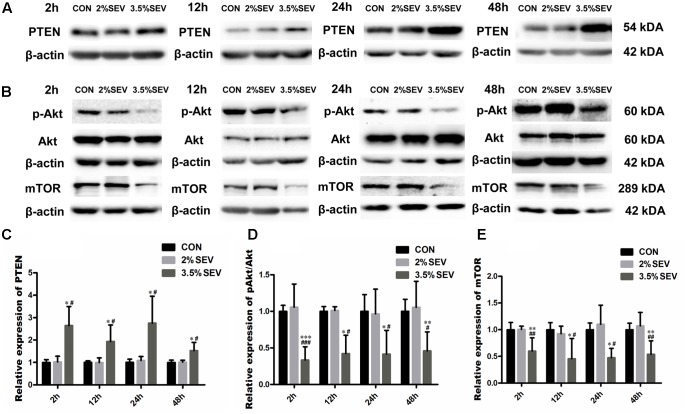
3.5% sevoflurane exposure upregualted autophagy via PTEN/Akt/mTOR pathway. 3.5% sevoflurane upregualted PTEN expression and inhibited p-Akt/Akt and mTOR expression in NSCs 2, 12, 24, 48 after anesthesia **(A,B)**. Quantification of PTEN **(C)**. Quantification of p-Akt/Akt **(D)**. Quantification of mTOR **(E)**. Values are presented as mean ± SD, *n* = 5; ^∗^*P* < 0.05, ^∗∗^*P* < 0.01, ^∗∗∗^*P* < 0.001 compared with the CON group, ^#^*P* < 0.05, ^##^*P* < 0.01, ^###^*P* < 0.001 compared with the 3.5%SEV group. One-way ANOVA with Newman–Keuls *post hoc* test or Kruskal–Wallis with Dunn’s Multiple comparison test was used for data analysis.

The results we demonstrated above was 3.5% sevoflurane upregulated the level of autophagy. However, the mechanism by which this occurred is unclear. To further investigate the mechanism of autophagy activation induced by 3.5% sevoflurane, we examined the PTEN/Akt/mTOR activity with Western blotting 2, 12, 24, and 48 h after gas exposure. 3.5% sevoflurane had significantly upregulated PTEN and downregualted p-Akt/Akt and mTOR in the fetal brains, compared to the CON and 2%SEV group (**Figure 3**, 3.5%SEV *vs.* CON: PTEN: *P* < 0.05, *P* < 0.05 *P* < 0.05, *P* < 0.05; p-Akt/Akt: *P* < 0.001, *P* < 0.05, *P* < 0.05, *P* < 0.01; mTOR: *P* < 0.01, *P* < 0.05, *P* < 0.05, *P* < 0.01; 3.5%SEV *vs*. 2%SEV: PTEN: *P* < 0.05, *P* < 0.05, *P* < 0.05, *P* < 0.05; p-Akt/Akt: *P* < 0.001, *P* < 0.05, *P* < 0.05, *P* < 0.05; mTOR: *P* < 0.01, *P* < 0.05, *P* < 0.05, *P* < 0.01; respectively). In accordance with the above findings, there was no difference between the 2%SEV and CON group.

### PTEN Inhibitor Attenuated Autophagy and Apoptosis and Enhanced NSC Proliferation after 3.5% Sevoflurane Anesthesia

**FIGURE 4:**
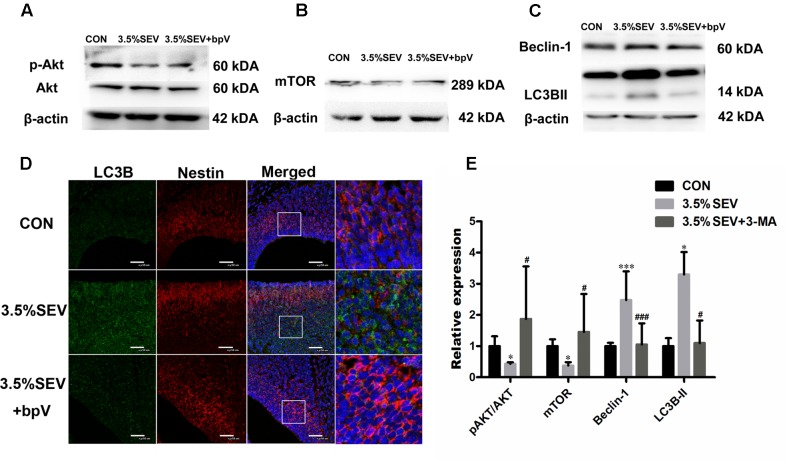
PTEN inhibitor alleviated sevoflurane-induced autophagy in the fetal brains. G14 rats were pretreated with bpV 1 h before sevoflurane exposure, expression of p-Akt/Akt and mTOR was upregulated **(A,B)**, and LC3BII and Beclin-1 expression was alleviated with bpV treatment **(C)**. LC3B (green) co-localized with Nestin (red) showed punctate pattern in the 3.5%SEV group, whereas both the CON group and 3.5%SEV + bpV group presented diffused green light labeling **(D)**. Scale bar = 50 μm. Quantification of p-Akt/Akt, mTOR, LC3BII and Beclin-1 **(E)**. Values are presented as mean ± SD, *n* = 5; ^∗^*P* < 0.05, ^∗∗^*P* < 0.01, ^∗∗∗^*P* < 0.001 compared with the CON group, ^#^*P* < 0.05, ^##^*P* < 0.01, ^###^*P* < 0.001 compared with the 3.5%SEV group. One-way ANOVA with Newman–Keuls *post hoc* test or Kruskal–Wallis with Dunn’s Multiple comparison test was used for data analysis.

**FIGURE 5:**
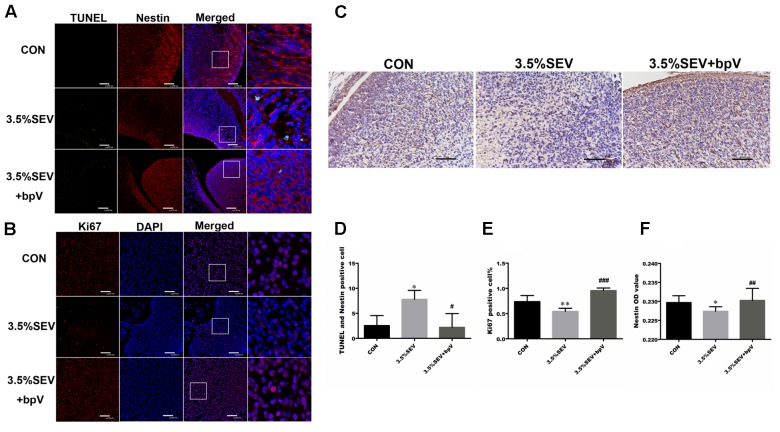
PTEN inhibitor alleviated sevoflurane-induced apoptosis and inhibited proliferation in the fetal brains. G14 rats were pretreated with bpV 1 h before sevoflurane exposure, NSC apoptosis was evaluated by TUNEL and Nestin immunofluorescence double staining **(A)**, and NSC proliferation was evaluated by Ki67 immunofluorescence **(B)** and Nestin immunohistochemistry **(C)**. Quantification of TUNEL/Nestin-positive cells **(D)**. Quantification of Ki67-positive cells **(E)**. Quantification of Nestin OD value **(F)**. Values are presented as mean ± SD, *n* = 5; ^∗^*P* < 0.05, ^∗∗^*P* < 0.01, ^∗∗∗^*P* < 0.001 compared with the CON group, ^#^*P* < 0.05, ^##^*P* < 0.01, ^###^*P* < 0.001 compared with the 3.5%SEV group. One-way ANOVA with Newman-Keuls *post hoc* test or Kruskal–Wallis with Dunn’s Multiple comparison test was used for data analysis. Scale bar = 50 μm.

In order to prove that autophagy was mediated by PTEN/Akt/mTOR pathway, the PTEN inhibitor, bpV, was intraperitoneally injected into G14 rats 1 h before gas treatment. Then the brains were harvested 24 h after anesthesia. The results showed p-Akt and mTOR levels were increased (**Figures 4A,B,E**, p-Akt/Akt: *P* < 0.05; mTOR: *P* < 0.05), and LC3BII and Beclin-1 levels were reduced in the 3.5%SEV + bpV group than the 3.5%SEV group (**Figures 4C,D,E**, LC3BII: *P* < 0.001; Beclin-1: *P* < 0.05). Besides the role of regulating autophagy, we also observed a decline in the number of TUNEL positive cells and an increase in both Ki67 positive cell rate and Nestin expression in the 3.5%SEV + bpV group compared with the 3.5%SEV group (**Figure 5**, TUNEL: *P* < 0.05; Ki67: *P* < 0.001; Nestin: *P* < 0.01), which indicated that autophagy might regulate apoptosis and proliferation when the fetal brains were exposed to 3.5% sevoflurane.

### Autophagy Inhibition Prevented Apoptosis of NSCs after Exposure of 2 h 3.5% Sevoflurane Anesthesia in E14 Fetuses

**FIGURE 6:**
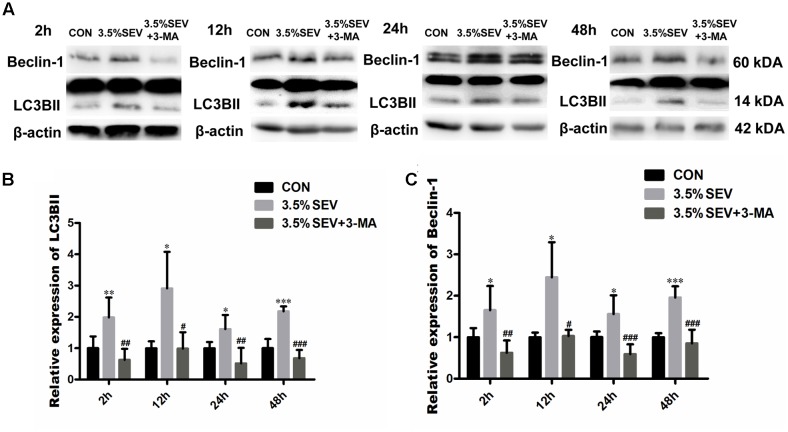
Autophagy inhibitor alleviated sevoflurane-induced autophagy in the fetal brains. G14 rats were pretreated with 3-MA. 2, 12, 24, and 48 h after sevoflurane exposure, NSC autophagy was evaluated by LC3BII and Beclin-1 Western blot **(A)**. LC3BII and Beclin-1 expression was alleviated with 3-MA treatment. Quantification of LC3BII **(B)** and Beclin-1 **(C)**. Values are presented as mean ± SD, *n* = 5; ^∗^*P* < 0.05, ^∗∗^*P* < 0.01, ^∗∗∗^*P* < 0.001 compared with the CON group, ^#^*P* < 0.05, ^##^*P* < 0.01, ^###^*P* < 0.001 compared with the 3.5%SEV group. One-way ANOVA with Newman–Keuls *post hoc* test or Kruskal–Wallis with Dunn’s Multiple comparison test was used for data analysis.

**FIGURE 7:**
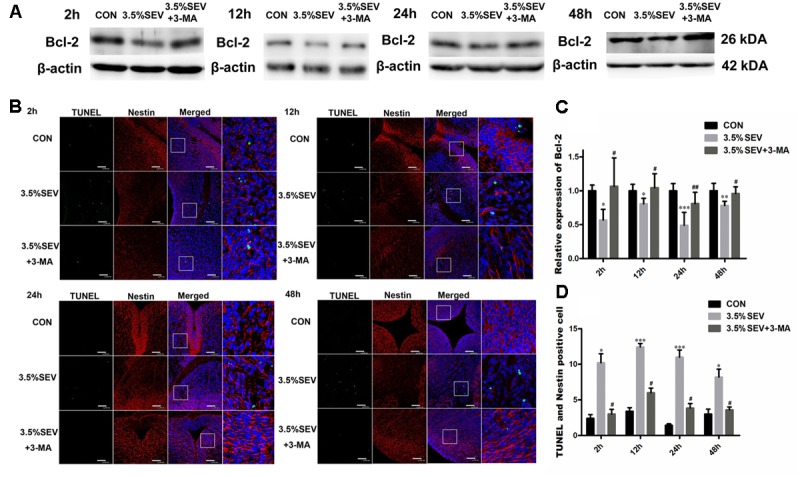
Autophagy inhibitor alleviated sevoflurane-induced apoptosis increase in fetal brains. G14 rats were pretreated with 3-MA. 2, 12, 24, 48 h after sevoflurane exposure, NSC apoptosis was evaluated by Bcl-2 expression **(A)** and immunofluorescence double staining of TUNEL and Nestin in the fetal brains **(B)**. Quantification of Bcl-2 expression **(C)**. Quantification of TUNEL/Nestin-positive cells **(D)**. Values are presented as mean ± SD, *n* = 5; ^∗^*P* < 0.05, ^∗∗^*P* < 0.01, ^∗∗∗^*P* < 0.001 compared with the CON group, ^#^*P* < 0.05, ^##^*P* < 0.01 compared with the 3.5%SEV group. One-way ANOVA with Newman–Keuls *post hoc* test or Kruskal–Wallis with Dunn’s Multiple comparison test was used for data analysis. Scale bar = 50 μm.

To reveal the effects of autophagy on NSC apoptosis, we injected 3-MA intraperitoneally into G14 rats 1 h prior to sevoflurane treatment. Then fetal brains were harvested at the four time points mentioned above. We evaluated the expression of LC3BII and Beclin-1 to verify the effects of 3-MA on the inhibition of autophagy. Both LC3BII and Beclin-1 levels were reduced as compared with the 3.5%SEV group (**Figure 6**, LC3BII: *P* < 0.01, *P* < 0.05, *P* < 0.01, *P* < 0.001; Beclin-1: *P* < 0.01, *P* < 0.05, *P* < 0.001, *P* < 0.001; respectively). Then we assessed the extend of apoptosis via with detecting TUNEL and Western blotting of the anti-apoptosis protein, Bcl-2. Exposure to 3.5% sevoflurane for 2 h induced NSC apoptosis in the immature brains with decreased Bcl-2 expression compared to the CON group (**Figures 7A,C**, Bcl-2: *P* < 0.05, *P* < 0.05, *P* < 0.001, *P* < 0.01; respectively). The Bcl-2 expression in the 3.5% sevoflurane plus 3-MA group was increased compared to the 3.5% sevoflurane group (**Figures 7A,C**, Bcl-2: *P* < 0.05, *P* < 0.05, *P* < 0.01, *P* < 0.05; respectively). TUNEL results also validated the anti-apoptotic effects of 3-MA on the developing brains exposed to 3.5% sevoflurane. More TUNEL-Nestin double positive cells were observed in the 3.5%SEV group than CON group (**Figures 7B,D**, TUNEL: *P* < 0.05, *P* < 0.001, *P* < 0.001, *P* < 0.05; respectively), and fewer positive cells were observed in the 3.5%SEV+3-MA group than 3.5%SEV group at all of the four time points (**Figures 7B,D**, TUNEL: *P* < 0.05, *P* < 0.05, *P* < 0.05, *P* < 0.05; respectively). Collectively, the results indicated that the hyperactive autophagy could lead to NSC apoptosis, and inhibiting autophagy could ameliorate NSC apoptosis.

### Autophagy Inhibition Enhanced NSCs Proliferation after Sevoflurane Anesthesia for 2 h in E14 Rats

**FIGURE 8:**
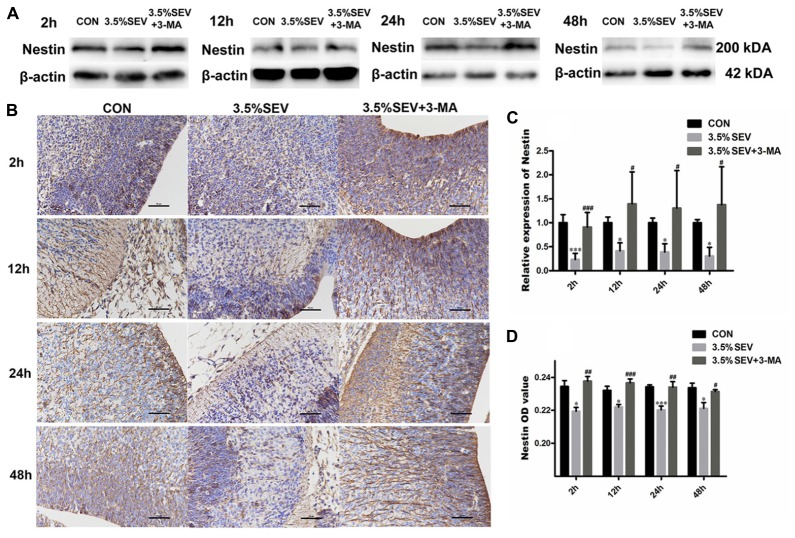
Autophagy inhibitor alleviated sevoflurane-induced proliferation inhibition in the fetal brains. G14 rats were pretreated with 3-MA. 2, 12, 24, and 48 h after sevoflurane exposure, NSC proliferation was evaluated by Nestin Western blot **(A)** and immunohistochemistry **(B)**. Expression of Nestin in the fetal brains **(C,D)**. Values are presented as mean ± SD, *n* = 5; ^∗^*P* < 0.05, ^∗∗^*P* < 0.01, ^∗∗∗^*P* < 0.001 compared with the CON group, ^#^*P* < 0.05, ^##^*P* < 0.01, ^###^*P* < 0.001 compared with the 3.5%SEV group. One-way ANOVA with Newman–Keuls *post hoc* test or Kruskal–Wallis with Dunn’s Multiple comparison test was used for data analysis. Scale bar = 50 μm.

**FIGURE 9:**
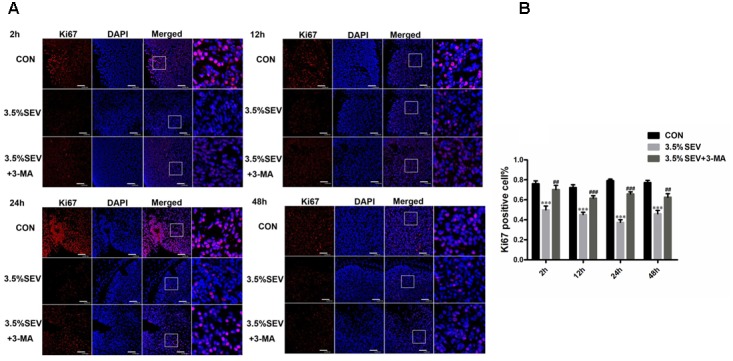
Autophagy inhibitor alleviated sevoflurane-induced proliferation inhibition in fetal brains. G14 rats were pretreated with 3-MA. 2, 12, 24, and 48 h after sevoflurane exposure, NSC proliferation was evaluated by Ki67 immunofluorescence **(A)**. Quantification of Ki67-positive cells in the fetal brains **(B)**. Values are presented as mean ± SD, *n* = 5; ^∗^*P* < 0.05, ^∗∗^*P* < 0.01, ^∗∗∗^*P* < 0.001 compared with the CON group, ^#^*P* < 0.05, ^##^*P* < 0.01, ^###^*P* < 0.001 compared with the 3.5%SEV group. One-way ANOVA with Newman–Keuls *post hoc* test or Kruskal–Wallis with Dunn’s Multiple comparison test was used for data analysis. Scale bar = 50 μm.

To examine whether autophagy was involved in the inhibited NSC proliferation, 3-MA was applied as described above in the G14 rats. Then we assessed the proliferation of NSCs by detecting Nestin expression with Western blotting and immunohistochemistry, and Ki67 expression with immunofluorescence staining. Exposure to 3.5% sevoflurane for 2 h disturbed NSC proliferation in the immature brains with a declined Nestin expression compared to CON group (**Figures 8A,C**, Nestin: *P* < 0.001, *P* < 0.05, *P* < 0.05, *P* < 0.05; respectively). The Nestin expression in the 3.5% sevoflurane plus 3-MA group was increased compared to the 3.5% sevoflurane group (**Figures 8A,C**, Nestin: *P* < 0.001, *P* < 0.05, *P* < 0.05, *P* < 0.05; respectively), which was consistent with the results of Nestin immunohisochemistry (**Figures 8B,D**, Nestin: 3.5%SEV *vs*. CON: *P* < 0.05, *P* < 0.05, *P* < 0.001, *P* < 0.05; 3.5%SEV + 3-MA *vs*. 3.5%SEV: *P* < 0.01, *P* < 0.001, *P* < 0.01, *P* < 0.05; respectively). Then we investigated the proliferation of NSCs with immunofluorescence staining. Neurogenesis in the fetal brain was evaluated by the ratio between Ki67 positive cells and total cells. We observed that Ki67 positive rate was decreased in the 3.5% SEV group compared to the CON group (**Figure 9**, Ki67: *P* < 0.001, *P* < 0.001, *P* < 0.001, *P* < 0.001; respectively), and the 3.5%SEV + 3-MA group presented a higher positive rate compared to the 3.5%SEV group (**Figure 9**, Ki67: *P* < 0.01, *P* < 0.001, *P* < 0.001, *P* < 0.01; respectively). The fundings above suggested that hyperactive autophagy could contribute to the descending proliferation of NSCs.

### Inhibition of Autophagy Alleviated Sevoflurane-Induced Neuronal Loss, Learning and Memory Impairment in the Offspring of Rats Anesthetized at G14

**FIGURE 10:**
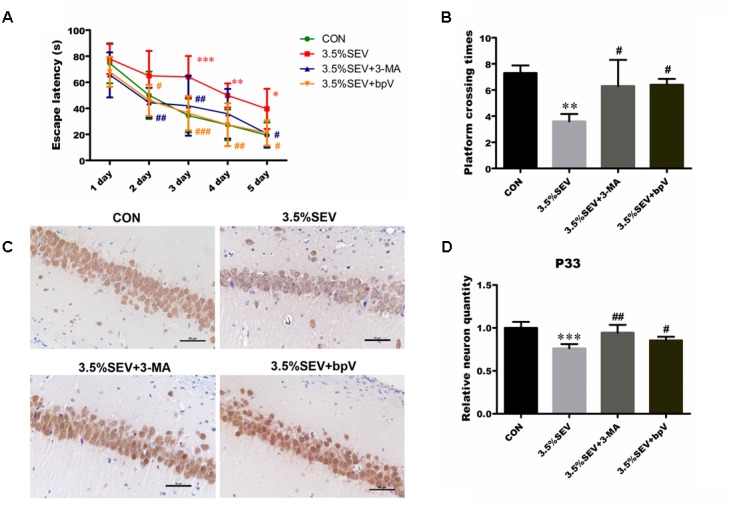
Inhibition of autophagy alleviated sevoflurane-induced neuronal loss and learning and memory impairment in the offspring of rats that were anesthetized at E14 day. Spatial cognitive performance was tested in the Morris water maze task 4 weeks after birth. The behavioral tests were evaluated by escape latency **(A)** and times across the platform **(B)**. Values are presented as mean ± SD, *n* = 10; ^∗^*P* < 0.05, ^∗∗^*P* < 0.01, ^∗∗∗^*P* < 0.001 compared with the CON group, ^#^*P* < 0.05, ^##^*P* < 0.01, ^###^*P* < 0.001 compared with the 3.5%SEV group. The data of escape latency were analyzed using two-way ANOVA followed by Bonferroni post test. The spatial probe test was performed at P33, and analyzed using Kruskal–Wallis with Dunn’s Multiple comparison test. Neurons in CA1 hippocampal region were valued by NeuN immunohistochemistry **(C)**. Quantification of relative NeuN-positive cells in the fetal brain **(D)**. Values are presented as mean ± SD, *n* = 5; ^∗^*P* < 0.05, ^∗∗^*P* < 0.01, ^∗∗∗^*P* < 0.001 compared with the CON group, ^#^*P* < 0.05, ^##^*P* < 0.01, ^###^*P* < 0.001 compared with the 3.5%SEV group. One-way ANOVA with Newman-Keuls *post hoc* test was used for data analysis. Scale bar = 50 μm.

We also explored the role of autophagy in learning and memory deficiencies by investigating the effect of 3-MA and bpV on neuron quantity in the hippocampal CA1 area and MWM performance. Pregnant rats delivered pups on G21 day. Learning and cognition of the offsprings were tested with the MWM on P28 to P33. There was no motor impairment among groups. We detected significant difference between groups during the learning phase using two-way ANOVA analysis (group: *P* < 0.0001; time: *P* < 0.0001). Consistent with previous findings, offsprings of the 3.5%SEV group took longer time to reach the platform as compared with the offsprings of the CON group from the third day of training. The application of 3-MA or bpV could significantly shorten the escape latency after the exposure of sevoflurane; there was no significant difference among else offsprings of the 3.5%SEV + 3-MA group, 3.5%SEV + bpV group and CON group (**Figure 10A**). During the spatial probe test, offspring from the 3.5%SEV group crossed the platform fewer times than offsprings from the CON group (**Figure 10B**, *P* < 0.01), 3.5%SEV + 3-MA group (**Figure 10B**, *P* < 0.05) and 3.5%SEV + bpV group (**Figure 10B**, *P* < 0.05), and there was no difference in 3.5%SEV + 3-MA group, 3.5%SEV + bpV group and CON group. After the spatial probe test, we assessed the quantity of neurons in CA1 hippocampal region by NeuN immunohistochemistry. The rats exposed to 3.5% sevoflurane for 2 h at E14 showed a declined relative neuron quantity compared with the CON group (**Figures 10C,D**, *P* < 0.001), the relative numbers of NeuN-positive cells in the 3.5%SEV + 3-MA and 3.5%SEV + bpV group were increased compared to the 3.5% sevoflurane group (**Figures 10C,D**, 3.5%SEV + 3-MA *vs.* 3.5%SEV: *P* < 0.01; 3.5%SEV + 3-bpV *vs.* 3.5%SEV: *P* < 0.05), and there was no difference compared with the CON group.

## Discussion

The extensive and growing use of anesthetics in pregnant women and young children raises significant concern regarding to their safety on developing brains (Zheng et al., 2013). According to the latest FDA warning, children under 3 years old or pregnant women in the third trimester who are exposed to anesthetics for more than 3 h or more than one exposure may experience adverse impacts on the brain development (Andropoulos and Greene, 2017). Numerous animal studies have been conducted during the synapse formation period, which occurs in P7 rodents and is the equivalent to the period from human late pregnancy to 3 years after birth (Jevtovic-Todorovic et al., 2003). Anesthetic-induced neurotoxicity in neonates is thought to be caused by increased neuronal apoptosis (Gentry et al., 2013). In contrast to other studies’ focusing on the synaptogenesis period, we considered the midtrimester a more pivotal period for brain development. With the rapid development of diagnostic and surgical techniques, there have been an emerging number of fetal *in utero* interventions in recent years. Midtrimester is a proper stage to perform these procedures as embryogenesis is then complete (Palanisamy, 2012). Nevertheless, the midtrimester is also a critical period of neurogenesis which is crucial to form a normal and intact nervous system in fetal brain. Massive neural stem/precursor cell proliferation happens in E14.5 rodents, which is equivalent to the brain development occurring during the midtrimester in human fetuses (de Graaf-Peters and Hadders-Algra, 2006; Palanisamy, 2012). In the same period, the fetal brain could be easily affected by external environment as well (Palanisamy, 2012). It has been reported that 3 and 4% sevoflurane anesthesia in G14 rats negatively affected learning and memory function in offsprings potentially via inhibition of proliferation and increase in apoptosis (Wang et al., 2017).

Given the FDA warning, anesthesia duration and frequency are considered key factors in anesthesia-induced neurotoxicity; however, the dose of the anesthetics may also play an important role (Lu et al., 2016). Compared to cesarean deliveries, fetal *in utero* interventions are usually performed with relative high-dose general anesthetics to prevent preterm labor and for a longer duration (Buck et al., 2008; Palanisamy, 2012). Since the exposure time (4–6 h) in rodent gestation is equivalent to 48 h anesthesia in a pregnant woman (Palanisamy, 2012), we speculated that a 2 h exposure in rats would be long enough to detect neurotoxic effects of sevoflurane. Our previous results showed 2% sevoflurane might not alter the normal central nervous system development in the fetus; however, 3.5% sevoflurane resulted in neurotoxicity as evidenced by increased apoptosis and reduced neurogenesis in E14 rats. Consistent with our findings, Wang et al. (2017) found both 3 and 4% sevoflurane resulted in neurotoxicity.

Besides apoptosis and neurogenesis, autophagy also participates in anesthesia-induced neurotoxicity. Autophagy is indispensable for normal brain development. In NSCs from E13.5-15.5 mice, the expression of autophagy related genes increase gradually (Vazquez et al., 2012). Interfering with the autophagic mechanism disturbs the proper orchestration of proliferation, differentiation and cell death necessary to establish the complex structure of central nervous system (Boya et al., 2008). Our results suggested that the autophagy level was increased when the E14 rats were exposed to 3.5% sevoflurane for 2 h. Consistent with our findings, Zhou et al. (2016) demonstrated that sevoflurane induced autophagy in H4 cells (Zhou et al., 2016). Similar to learning and memory dysfunction induced by maternal anesthesia, ethanol consumption during pregnancy may also result in abnormal development and behavior in offsprings, known as fetal alcohol spectrum disorders (FSAD). Ethanol could damage the mitochondria in neonatal rat neurons, trigger production of ROS, and thus activate autophagy, which may be mediated by downregulation of mTOR signaling pathway (Chen et al., 2012). General anesthetics can also increase the production of ROS, whose important roles in autophagy have been widely documented (Scherz-Shouval and Elazar, 2011; Vernon and Tang, 2013). Collectively, the above evidence indicates that sevoflurane can lead to the activation of autophagy.

To our knowledge, this study is the first to provide the evidence of NSC autophagy in the fetus exposed to sevoflurane, and our results indicated that autophagy was regulated via the PTEN/Akt/mTOR pathway. And the effect could be alleviated by PTEN inhibitor bpV. The PI3K/Akt/mTOR signaling pathway has been reported to be an important negative regulator of autophagy (Heras-Sandoval et al., 2014). PTEN can regulate autophagy in mammalian cells by antagonizing PI3K through its lipid phosphatase activity (De Amicis et al., 2014). Moreover, PTEN can also affect the brain development as suggested by its abundant expression in embryonic central nervous system (Gimm et al., 2000). As a tumor suppressor, PTEN could negatively regulated the proliferation of neural stem/progenitor cells in E14.5 mice, and its deletion could also alleviate apoptosis (Groszer et al., 2001). In addition to reduced autophagy by inhibition of PTEN, declined apoptosis and enhanced neurogenesis in 3.5%SEV + bpV group were also observed. Inhibition of PTEN was reported to reduce neuronal apoptosis after oxygen glucose deprivation (OGD) and meliorate neuronal polarity (Zhao et al., 2013). Besides neurogenesis and apoptosis, learning and memory function were the most worrying. We found in this study that mTOR, which is critical for memory consolidation, storage and retrieval processes (Neasta et al., 2014), was downregulated in the NSCs from offspring of the group treated with 3.5% sevoflurane. All the cues suggested a complex interaction between autophagy, apoptosis, proliferation and spatial learning and memory, which contributed to the neurotoxic effect of sevoflurane.

The relationship between sevoflurane anesthesia-induced apoptosis and autophagy in the NSCs remains poorly understood. Both apoptosis and autophagy participate in development of the central nervous system. During brain development, apoptosis is the main mechanism to prevent excessive cell survival (Cecconi et al., 2008). Our results demonstrated that 3.5% sevoflurane-induced autophagy enhanced apoptosis of NSCs, and inhibition of autophagy with 3-MA reduced apoptosis. Basal autophagy may be a survival mechanism that reduces the accumulation of abnormal proteins and promotes turnover of damaged organelles and recycling energy and nutritions. Zhou et al. demonstrated that 4.1% sevoflurane reduced ER stress and activated autophagy, which protected H4 cells from sevoflurane-induced apoptosis (Zhou et al., 2016). Such discrepancy could be due to that autophagy level may vary with the cell types as well as the concentration, duration and timing of anesthetic exposure. And 3.5% sevoflurane could induce excessive autophagy (Yang and Wei, 2017). Overactive autophagy may impair the basic cellular functions or even directly promote cell death by inducing excessive degradation of critical cellular constituents, depleting cellular energy and disturbing the intracellular environmental (Liang, 2010). Due to extensive production of damaged organelles, anesthesia-induced overwhelming autophagic stress is responsible for cell death (Orrenius et al., 2011). It has been reported that autophagy is critical for the initiation and execution of apoptosis, presumably through common regulators such as BCL2 family proteins. Moreover, autophagosomes provide caspase processing with a membrane-based platform within the cell to promote apoptosis (Young et al., 2012). A previous study showed that insulin withdrawal resulted in the increase of autophagic flux and cell death, which could be alleviated by inhibiting autophagy and aggravated with the rapamycin to promote autophagy (Yu et al., 2008). Another study indicated that MK801 induced blockade of the NMDA receptor promoted autophagic pathways, which preceded apoptosis in immature GABAergic interneurons, and the use of 3-MA could prevent apoptosis (Roux et al., 2015). The results above provided basestone to speculate that the autophagy might promote apoptosis.

It has been proved that neuroapoptosis is not the only reason to be responsible for developmental anesthesia related neurotoxicity (Yu et al., 2017). After exposure to 3.5% sevoflurane, NSCs in fetal brains exhibited decreased proliferation. In the present study, we found the increased autophagy also contributed to the proliferation decline as pharmacological inhibition of autophagy with 3-MA markedly improved the expression of Nestin and the ratio of Ki67 positive cells. It has been reported that disruption of the autophagic machinery can alter proper proliferation. Ambra1 (autophagy and beclin 1 regulator 1) is necessary to control cell proliferation, and Beclin-1 is negatively associated with cell proliferation (Liang et al., 1999; Fimia et al., 2007; Cecconi et al., 2014). Furthermore, loss of Atg5 in the cortex specifically resulted in increased neuronal proliferation (Lv et al., 2014). These data support the conclusion that the high levels of autophagy caused by sevoflurane may be partially responsible for the inhibition of NSC proliferation.

Apart from abnormal neurogenesis and neuroapoptosis, other factors like autophagy might also lead to adverse neurocognitive sequelae. However, there was rare evidence on the relationship between autophagy and sevoflurane induced cognitive dysfunction. Contrary to the our results that inhibition of autophagy alleviated cognitive dysfunction, blocking autophagy by chloroquine exacerbated sevoflurane induced neuronal apoptosis and memory impairment (Zhang et al., 2016). This discrepancy could be explained by the different mechanisms of autophagy inhibition by chloroquine and 3-MA.

Our study does have a few limitations. First, we did not observe dynamic changes of autophagy over time. Instead, we compared the changes in autophagy levels in different treatment groups; also, in terms of clinical applications, general anesthesia usually does not involve only one single general anesthetic agent. Combined use of general anesthetics may perplex the neurotoxicity outcome. Further studies on the effect of combined use of anesthetic agents are necessary to elucidate the issue.

In summary, our research demonstrated that autophagy was upregulated through the PTEN/Akt/mTOR pathway when the E14 rats were exposed to 3.5% sevoflurane for 2 h, and that the activated autophagy led to increased apoptosis, declined NSCs proliferation, decreased relative neuron quantity, and impaired neurocognitive function. Understanding the role of NSC autophagy induced by 3.5% sevoflurane may provide novel strategies to alleviate the neurodevelopment impairment in the future.

## Author Contributions

XL and PZ designed the experiments. XL, ZW, and PZ contributed to the planning of the work. XL performed all the experiments with the help of ZW, YZ, YX, and GH. ZW, YZ, YX, and GH participated in the data collection. XL, YZ, YX, and GH analyzed and interpreted the results. XL wrote the manuscript with the help of YX and GH. PZ supervised the project and revised the article.

## Conflict of Interest Statement

The authors declare that the research was conducted in the absence of any commercial or financial relationships that could be construed as a potential conflict of interest.
